# A Network Topology Control and Identity Authentication Protocol with Support for Movable Sensor Nodes

**DOI:** 10.3390/s151229782

**Published:** 2015-12-01

**Authors:** Ying Zhang, Wei Chen, Jixing Liang, Bingxin Zheng, Shengming Jiang

**Affiliations:** 1 College of Information Engineering, Shanghai Maritime University, Shanghai 201306, China; yingzhang@shmtu.edu.cn (Y.Z.); liangjixing501@163.com (J.L.); zhengbingxin501@163.com (B.Z.); smjiang@shmtu.edu.cn (S.J.); 2 Department of Computer Science, Tennessee State University, Nashville, TN 37209, USA

**Keywords:** wireless sensor network, nodes mobility, security topology control, identity authentication, soft handover

## Abstract

It is expected that in the near future wireless sensor network (WSNs) will be more widely used in the mobile environment, in applications such as Autonomous Underwater Vehicles (AUVs) for marine monitoring and mobile robots for environmental investigation. The sensor nodes’ mobility can easily cause changes to the structure of a network topology, and lead to the decline in the amount of transmitted data, excessive energy consumption, and lack of security. To solve these problems, a kind of efficient Topology Control algorithm for node Mobility (TCM) is proposed. In the topology construction stage, an efficient clustering algorithm is adopted, which supports sensor node movement. It can ensure the balance of clustering, and reduce the energy consumption. In the topology maintenance stage, the digital signature authentication based on Error Correction Code (ECC) and the communication mechanism of soft handover are adopted. After verifying the legal identity of the mobile nodes, secure communications can be established, and this can increase the amount of data transmitted. Compared to some existing schemes, the proposed scheme has significant advantages regarding network topology stability, amounts of data transferred, lifetime and safety performance of the network.

## 1. Introduction

Mobile wireless sensor networks (MWSNs) are a kind of wireless sensor network. Usually they are composed of any minority or majority of mobile sensor nodes in a monitoring area, and they can form an arbitrary network topology among the nodes. The main difference with a static wireless sensor network is the mobility support of the nodes. After one-time deployment of a static sensor network, the positions of nodes are fixed, and centralized control can be realized. However in the MWSN, usually there are some nodes joining and exiting the network, in addition, the mobility of nodes makes the deployment area dispersive, so only distributed control can be realized [1,2]. MWSNs not only have the characteristics of traditional WSNs, but also have their own advantages, which makes them have wide application prospects in military applications, environmental monitoring, medical care and other related fields [3]. On the other hand, the mobility of nodes also brings some problems. The topology of the network changes frequently and the communication links are not stable, which can interrupt the data transmission or decrease the amount of data transmitted. The identity authentication after movement and the large energy consumption are the main bottleneck for the application of MWSNs. Therefore, research on an efficient topology control algorithm for MWSNs has great significance [4,5]. It can make nodes adjust their own working state according to the environment changes and adapt to the dynamic environment, so it can make the WSN support node movement better, and also extend the lifetime of the network, and improve the communication capacity [6].

Aiming at addressing some of the problems of MWSNs, like the frequent topology changes, large energy consumption and security weakness, a kind of efficient Topology Control algorithm support for nodes Mobility (TCM) is proposed in this paper. A Particle Swarm Optimization (PSO) clustering algorithm based on multi-objective optimization was used to solve the problems of uneven clustering and energy consumption in the topology construction phase. In the topology maintenance stage, aiming at the mobility of the nodes, a digital signature authentication method based on Error Correction Code (ECC) and the soft handover communication mechanism were adopted. This can increase the amount of data transmitted and extend the network lifetime on the basis of considering the establishment of secure communication between the nodes.

The structure of the rest of the paper is as follows: Section 2 introduces some work related to the research and compares several classical algorithms for topology control. Section 3 describes the TCM method in detail, and in Section 4, some simulations have been carried out and the TCM method is compared with some other methods. Finally, Section 5 concludes this paper. 

## 2. Related Work

Most of the energy consumption in a WSN derives from the data transmission, and lots of research shows that the network can better support node movement, improve network coverage and extend the network lifetime by using some form of clustering technology [7]. The use of clustering technology was first put forward in WSNs with the Low-Energy Adaptive Clustering Hierarchy (LEACH) protocol [8]. In the algorithm, the cluster head is elected by the circulation mode, and every round is divided into two parts, which are cluster formation and stable operation. In the cluster formation stage, the sensor nodes in the monitoring area randomly generate a number within the range of [0, 1], and if the number is less than the threshold *T*(*n*), then the sensor node will be selected as the cluster head. The cluster head broadcasts the information and the sensor nodes choose their clusters according to the strength of the received signal, and finally the clustering for the monitoring area is completed. In the stable operation stage, the cluster head will deal with the information from sensor nodes and send the messages to the base station, and then start the next round of clustering until the energy is exhausted. In this method, the sensor nodes do not transmit data in multi-hops, and this can decrease the energy consumption in data transmission, but the selection of cluster heads is random, so it cannot realize uniform clustering, and it does not consider the factor of energy of the nodes, which may cause the early death of cluster heads. Actually, the cluster head selection is just suitable for static networks, and it does not apply to the movable nodes in MWSNs. 

Many researchers have proposed numerous improvements for LEACH, like Low-Energy Adaptive Clustering Hierarchy for Mobile nodes (LEACH-M) [9], which is an improved topology algorithm for node mobility. In the cluster head selection, it considers the mobility of the nodes and uses a wake mechanism based on a Time Division Multiple Address (TDMA) schedule. This allows the nodes to remain dormant in the TDMA time slot which is not theirs, so it can decrease the energy consumption. However the waking mechanism is complex, and it needs at least three TDMA frames for the mobile nodes to be rediscovered by the cluster head and establish a communication link with the cluster head. This may cause some parts of the transmitted data to be lost. The Adaptive LEACH-Mobile (ALM) algorithm [10] introduces the mobility metric on the base of LEACH-M, which considers both the mobility and energy consumption, and it improves the wake mechanism. When the sensor nodes do not receive data from the cluster head for two continuous TDMA time slots, they will automatically increase the communication range. This method maintains the links between sensor nodes and cluster head by increasing the communication range, so it will increase relatively the communication overhead of cluster heads.

When the clustering is stable, it is necessary to consider the safe establishment of communication links between the sensor nodes and cluster head, which is a key step for data collection and transmission. Currently, there are not many research achievements on network security with changing topology, and most of these research achievements are based on static networks. Reference [11] proposed a new key management scheme for mobile heterogeneous WSNs based on asymmetric key pre-distribution and hash functions. It uses a seed key and hash function to realize the authentication of a mobile cluster head, but it only permits cluster heads move, and all the member nodes are static. Aiming at addressing the disadvantages of the Localized Encryption and Authentication Protocol (LEAP) [12] and Opaque Transitory Master Key (OTMK) [13], reference [14] helped the mobile nodes join the network safely in a mobile scenario by establishing pair keys and local broadcast keys with the new neighbor nodes. In [15,16], researchers introduced the elliptic curve into the public key in the key management mechanism, and its security is a discrete logarithm problem based on elliptic curve. On the premise of the same security indexes, the length of the key in ECC [17] is shorter than those used in other public key mechanisms, so it needs less computation and storage space and it has been regarded as an encryption algorithm with the highest encryption intensity in the existing public key cryptosystem [18]. It can effectively verify the identity of the mobile nodes and the new nodes to ensure the network security.

## 3. TCM Protocol

### 3.1. Network Model and Hypothesis

**Figure 1:**
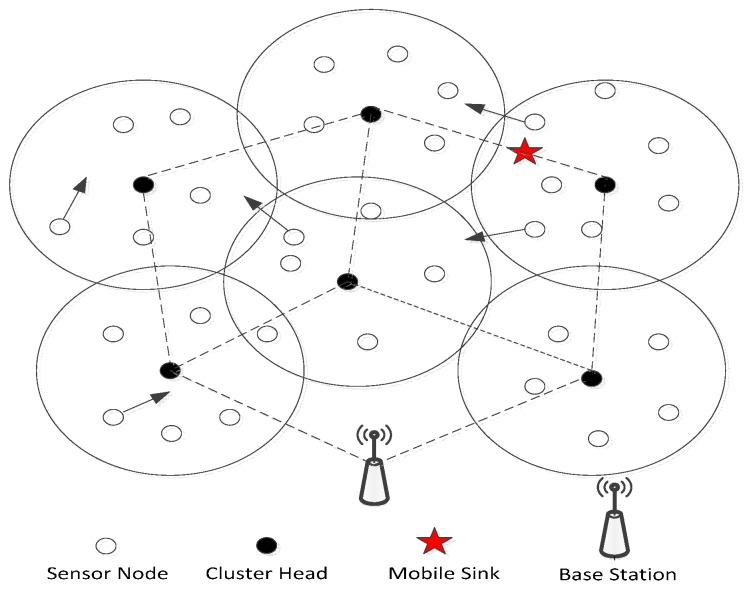
The schematic model of the network.

The schematic network model in this paper is shown as Figure 1. Marine environmental monitoring is its application background. In the monitoring area, nodes on the sea surface may move with different speed and direction in a certain range under the impulse of the waves and winds. The mobile monitoring ship is just like the mobile sink in the network system. Since the mobile WSN has totally different features from a static WSN, and it is relatively more complex, for discussion convenience, we pose some hypothesis for the network model:

1. The physical properties for all the nodes like the configurations of energy and hardware are the same.
2. All the nodes are on the same plane and their original positions are random.
3. All the nodes can move in the monitoring area with a random speed and direction.
4. The sink nodes have more abundant resources than the ordinary sensor nodes, such as communication range and storage space.

The mobile sink moves in the network and communicates with the nodes in its communication range, and it will collect the data fused by cluster heads along the planned route. It takes on the topology establishment and maintenance tasks. The base station is fixed and it communicates with the cluster heads, in fact, it can transmit the information collected by the sensor nodes to the control center but the mobile sink cannot.

### 3.2. The Topology Establishment Phase 

**Figure 2:**
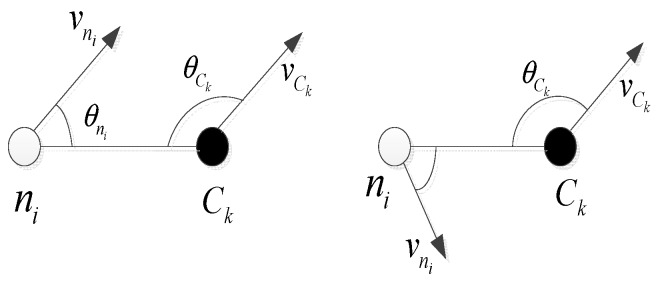
The mobility of the nodes.

Currently most of the clustering algorithms such as the LEACH-M and ALM just improve the selection of cluster heads on the basis of LEACH. These algorithms do not consider certain factors like energy, distance, position and mobility which can likely influence the clustering results. Meanwhile, the cluster head selection algorithm itself has the feature of randomness, so it cannot ensure the stability of the number of cluster heads and a balanced distribution. This may cause more energy consumption and isolated nodes. To solve these problems, we put forward an efficient clustering algorithm support for mobile nodes. On the basis of the algorithm described in [19], we consider the factors like energy, distance and mobility comprehensively to ensure the sum of the distances between the sensor nodes and cluster head is minimal and the energy left is maximal. In addition, the node with the minimal moving factor will be the cluster head. All these intend to solve the problem of unbalanced clustering and the mobility of the nodes. The fitness function is defined as follows:

(1) $$F=a_{1}*f_{1}+a_{2}*f_{2}+(1-a_{1}-a_{2})*f_{3}$$

(2) $$f_{1}=\underset{k=1,2\cdots K}{\max}\left\{\sum_{\forall n_{i}\in C_{k}}d(n_{i},C_{k})/Num_{C_{k}}\right\}$$

(3) $$f_{2}=\sum_{i=1}^{N}E(n_{i})/\sum_{k=1}^{K}E(C_{k})$$

(4) $$f_{3}=\underset{k=1,2,...K}{\max}\left\{\sum_{\forall n_{i}\in C_{k}}\sqrt{v_{n_{i}}^{2}+v_{C_{k}}^{2}-2v_{n_{i}}v_{C_{k}}\cos(\frac{\theta_{n_{i}}+\theta_{C_{k}}}{2})}\right\}$$

where, $f_{1}$ is the maximal average Euclidean distance between the sensor nodes and the cluster head. $d(n_{i},C_{k})$ is the distance between the node $n_{i}$ and its cluster head. *Num_c_k__* is the number of nodes in the cluster $C_{k}$. $f_{2}$ is the ratio of the sum of all the nodes’ and all the cluster heads’ energy. $f_{3}$ is the movement factor, and it only represents the relative movement of the sensor nodes and cluster head, not the sum of vector of velocities. $v_{C_{k}}$ is the velocity of the cluster head, and $v_{n_{i}}$ is the moving velocity of ordinary sensor nodes. $\theta_{n_{i}}$ is the movement angle of sensor nodes and, $\theta_{C_{k}}$ is the movement angle of the cluster head. The mobility of the nodes is shown in Figure 2.

The clustering algorithm runs as follows:

(1) Initialize the position and velocity of particle P(P=1,2,⋯S) Each has *K* candidate cluster heads.
(2) Assign node *n_i_* to the closest cluster head, calculate the fitness values of each particle P(P=1,2,⋯S) using Equations (1)–(4).
(3) Select the global optimal extremum *G*(*t*) of the population and the individual optimal extremum *P_i_*(*t*) of each particle.
(4) Update the position and velocity of particles.
(5) Mapping the position of particle to the actual cluster head.
(6) Repeat steps (1) to (5) until the maximum iteration is reached or the required coverage rate is satisfied.

**Figure 3:**
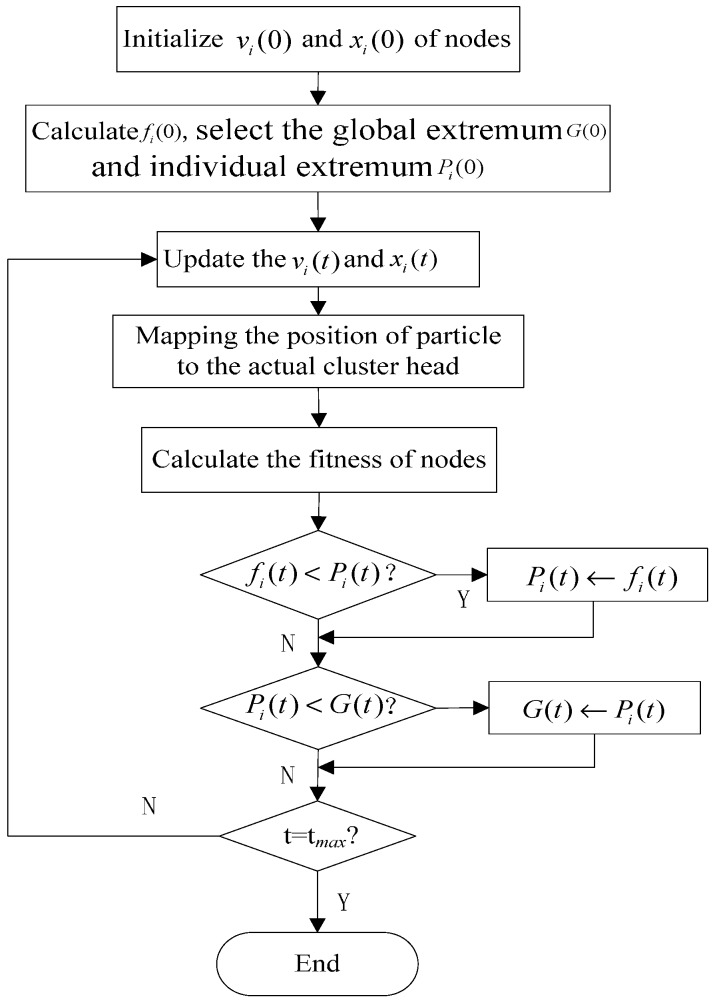
The clustering flowchart.

The clustering process is shown in Figure 3. After clustering, the network is in the topology maintenance phase. Sensor nodes will transmit the data to cluster heads by a single-hop transmission mode. 

### 3.3. The Topology Maintenance Phase 

After clustering, the network will be in the topology maintenance phase. The sensor nodes will establish a safe communication link with a cluster head, and then send the collected data to the cluster head. The mobile sinks will collect the data fused by cluster heads along with the planed route. Because of the mobility of the nodes, the topology may change. The three main reasons are as follows: the first is the nodes’ movement within the cluster, the second is the nodes’ movement between the different clusters, and the last is that some nodes will occasionally join in or die. Malicious nodes can also forge legal nodes, and obtain sensitive network information. In order to avoid this happening, we must perform identity authentication for the nodes after their dynamic changes. Besides considering the security factor, the energy consumption and efficient transmission of data are also important factors which should be seriously considered.

Before the nodes’ deployment, the server creates a couple of keys for each node, namely the public key P and private key S, and they satisfy the equation P=S∗G, where *G* is the base point on the elliptic curve. Each sensor node stores a unique identity $ID_{N_{i}}$, a couple of keys ($S_{N_{i}}$,$P_{N_{i}}$), digital signature $\left(W_{N_{i}},w_{N_{i}}\right)$, binary symmetric function *F*, and the hash function *H*. The mobile sink restores the only identity $ID_{MS}$ of the whole network, a couple of keys ($S_{MS}$,$P_{MS}$), digital signature $\left(W_{MS},w_{MS}\right)$ and the hash function *H*. The expression of the digital signature $\left(W_{i},w_{i}\right)$ is shown as follows:

(5) $$\left\{ \begin{array}{l} W_{i}=r_{i}\times G=(x_{w_{i}},y_{w_{i}})\\ w_{i}={r_{i}}^{-1}(H(ID_{i}||T_{i})+S\times x_{w_{i}})(mod\;n) \end{array}\right.$$

where $r_{i}$ is a random number. G is the base point on the elliptic curve. *H* is the hash function, and S is the private key. 

If node i sends a verification request for the digital signature $\left(W_{i},w_{i}\right)$ within time T, we can prove the legitimacy of node i only if we prove the following equation $N_{i}=W_{i}$ is true. The process is shown as follows:

(6) $$\begin{array}{l} N_{i}=w_{i}^{-1}H(ID_{i}||T)G+w_{i}^{-1}x_{w_{i}}P\\ \text{ =}w_{i}^{-1}(H(ID_{i}||T)+x_{w_{i}}S)G\\ \text{ =}w_{i}^{-1}r_{i}r_{i}^{-1}(H(ID_{i}||T)+x_{w_{i}}S)G\\ \text{ =}w_{i}^{-1}r_{i}\{r_{i}^{-1}(H(ID_{i}||T)+x_{w_{i}}S)\}G\\ \text{ =}w_{i}^{-1}r_{i}w_{i}G\\ \text{ =}W_{i} \end{array}$$

#### 3.3.1. Movement of the Nodes within a Cluster

**Figure 4:**
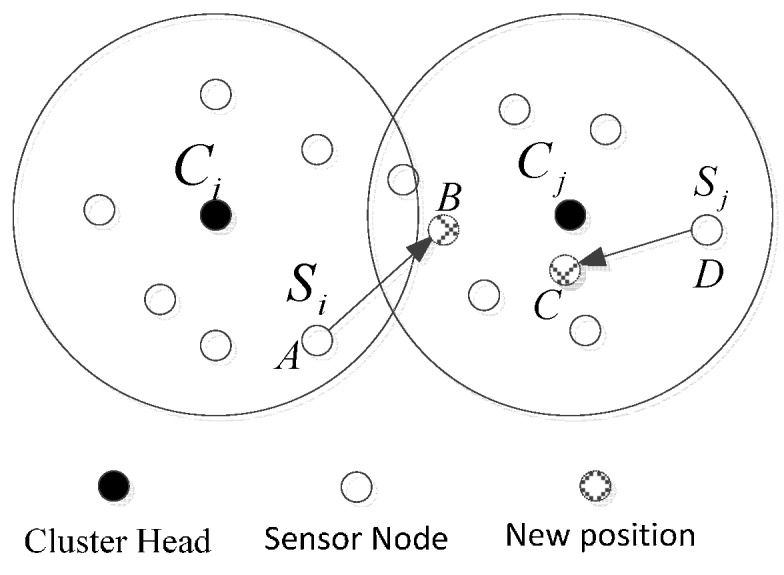
A schematic description of nodes’ mobility in a sensor network.

After entering the topology maintenance phase, for the purpose of saving communication energy consumption, the sensor nodes only upload the data in their own TDMA time plots. In other time plots, they do not communicate with any neighbor nodes, and at this time, they will be in sleep mode and wait to be awakened in the next round. In the public time plot of the member nodes, the nodes do not send messages to cluster heads directly, but wait for a data transmission request Data_req from a cluster head. The nodes will transmit the data to a cluster head only after they receive Data_req. If the movement of some sensor nodes has happened within the cluster, it can be described by the schematic of Figure 4. $S_{j}$ moves from position *D* to *C* in cluster $C_{j}$, and cluster head broadcasts the request Data_req in its time plot. Because it moves within the cluster, the nodes can receive the request and upload the collected data. The schematic description of nodes’ mobility in a sensor network is shown in Figure 4.

#### 3.3.2. Nodes Moving between Clusters

When the nodes move between different clusters, the communication mechanism of soft handover was adopted to keep the continuity of data transmission. When the node moves to cross the boundary of the clusters, this mechanism can keep the node communicate with the new and the old cluster heads at the same time, and when it establishes a stable link with the new cluster, it will cut off the link with the old cluster head. As shown in Figure 4, the node *S_i_* moves from cluster *C_i_* to *C_j_*, and from position A to B. In the time slot *T_i_* of *S_i_*, *C_i_* broadcasts the request *Data_req*. Since *S_i_* has already moved to *Cj*, *C_i_* cannot receive the data uploaded by *S_i_*. In this case, *C_i_* signs *S_i_* as a pseudo-mobile node, and cluster head *C_i_* increases the communication range adaptively to keep the link with *S_i_*. On the other hand, *S_i_* also cannot receive *Data_req* in time slot *T_i_*, it signs itself as a pseudo-mobile node, and broadcasts the *Join_req*: *S_i_*→*:*Join_req*║*ID_Si_*║*P_Si_*║(*W_Si_*,*w_Si_*) to request joining the cluster *Cj*. The cluster *Cj* makes an identity authentication to *S_i_* by using the digital signature (*W_Si_*,*w_Si_*), and then it gives the response information *ACK*: *C_i_*→*S_i_*:*ACK*║*ID_Ci_*║*P_Ci_*║(*W_Ci_*,*w_Ci_*). Then the cluster *Cj* updates the member list of cluster, assigns a new time plot to *S_i_*, and broadcasts this within the cluster. When the node *S_i_* receives the new TDMA frame, confirms itself as a mobile node, and sends a quit request *Exit_req* to the original cluster *C_i_*. After cluster head *C_i_* receives the quit request *Exit_req*, it will sign *S_i_* as a mobile node, and delete the time plot of *S_i_* and other relative key information.

#### 3.3.3. New Nodes Join in or Node Death

When the node $S_{i}$ joins in a cluster, it will broadcast the join request information, the pre-stored identity *ID_Si_*, the public key *P_Si_,* and the digital signature (*W_Si_*,*w_Si_*): *S_i_*→*:*Join_req*║*ID_Si_*║*P_Si_*║(*W_Si_*,*w_Si_*). After the current cluster head *C_i_* verifies its legitimacy by (*W_Si_*,*w_Si_*), it will give the response answer *C_i_*→*S_i_*:*ACK*║*ID_Ci_*║*P_Ci_*║(*W_Ci_*,*w_Ci_*). On the other hand, after the node *S_i_* verifies the legitimacy of the cluster head by (*W_Ci_*,*w_Ci_*), *S_i_* will join in cluster *C_i_*, and establish the session key with *C_i_*. *C_i_* assigns a time plot to *S_i_*, and broadcasts this within the cluster. The topology control protocol is a dynamic clustering algorithm, the energy factor of the nodes has been considered in the clustering process. When there are dead nodes in network, they are not allowed to participate in the clustering and they will be excluded by the network.

## 4. Simulation and Evaluation

We use MATLAB R2009a to carry out the simulation, and in this paper the simulation parameters are set as follows: 100 nodes are randomly deployed in a region of 100 m × 100 m. The base station locates on the point (50, 50). The original energy of the nodes is 0.1 J. The speed of movement of the nodes is in the range of 0-10 m/s and the angle of movement is in the range of [0, 180°]. The number of cluster heads is six (the base station is regarded as a fixed cluster head). The weight coefficients are *a*_1_ = 0.3, *a*_2_ = 0.3. The length of data transmitted for each round is 4000 bit. The energy model is adopted as in [20].

When transmitting *K* bit data, and the distance is *d*, the energy communication of a node can be calculated as follows:

(7) $$\begin{array}{l} E_{Tx}\text{( }k\text{,}d\text{ ) = }E_{Tx\_elec}\text{(}k\text{) + }E_{Tx\_amp}\text{(}k\text{,}d\text{)}\\ \text{ =}\left\{ \begin{array}{l} kE_{elec}\text{+ }k\varepsilon_{fs}d^{2}\;d\text{<}d_{0}\\ kE_{elec}\text{+ }k\varepsilon_{mp}d^{4}\;d\geq d_{0} \end{array}\right. \end{array}$$

where the $\varepsilon_{fs}$ and $\varepsilon_{mp}$ are energy consumption coefficients of the power amplifier circuit, and $d_{0}=\sqrt{\varepsilon_{fs}/\varepsilon_{mp}}$.

The energy consumed by receiving *K* bit data is calculated as follows:

(8) $$E_{Rx}(k)=E_{Rx\_elec}(k)=kE_{elec}$$

where *E_elec_* = 50 nJ/bit, *ε_fs_* = 10 pJ/bit/m^2^ and *ε_mp_* = 0.00013 pJ/bit/m^4^. The energy consumption of data fusion is *E_DA_* = 5 nJ/bit.

### 4.1. Network Stabilization

**Figure 5:**
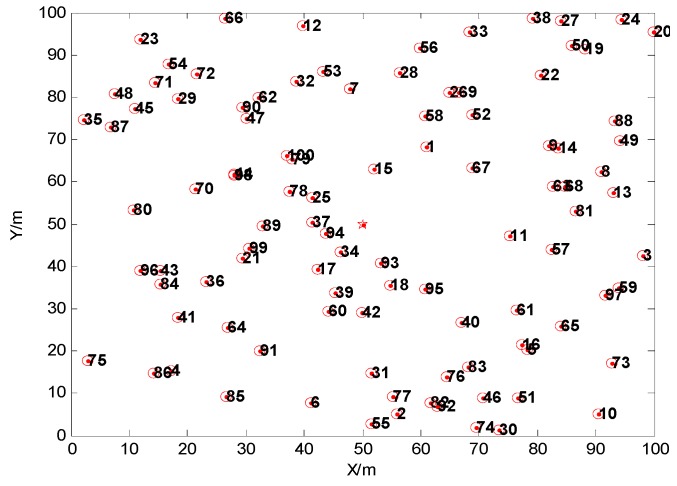
The original random deployment of the nodes.

**Figure 6:**
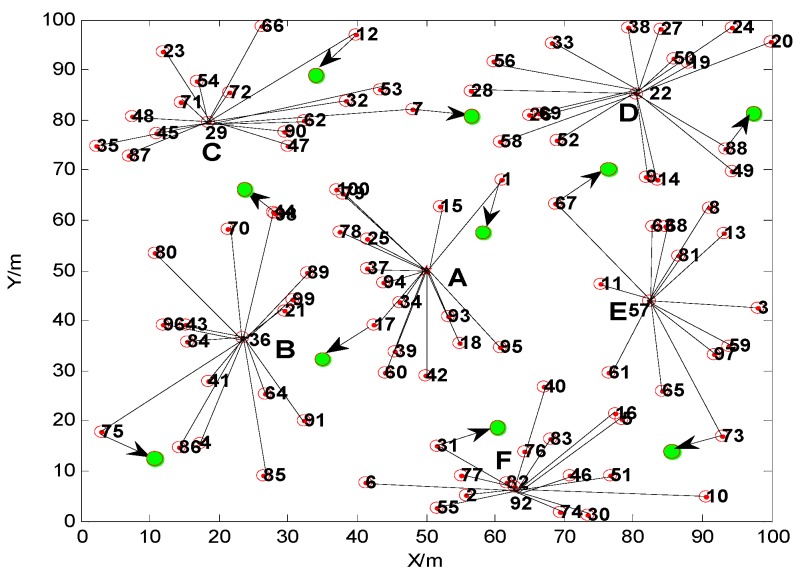
The structure of network after the movement of 10% of the nodes.

**Figure 7:**
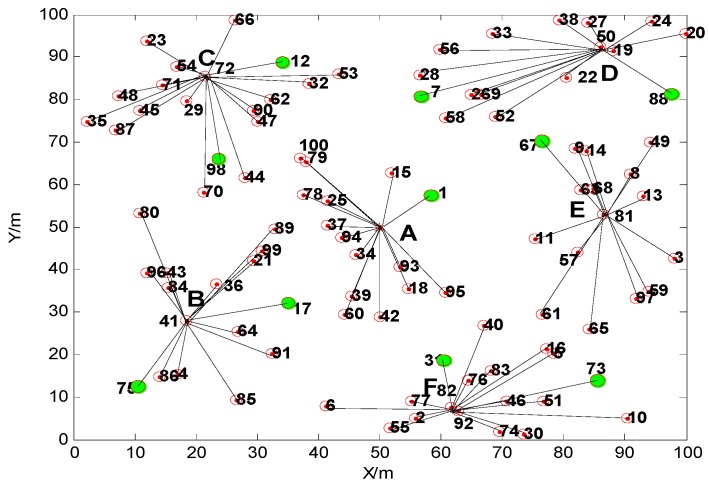
The clustering topology of network for a new round after nodes’ movement.

The original random node deployment is shown in Figure 5, the structure of the network after the movement of 10% of the nodes is shown in Figure 6, and the clustering topology of the network for a new round after the nodes’ movement is shown in Figure 7.

Comparing Figure 6 and Figure 7, we can find that the movement of the nodes has a great influence on the network’s topology. Not only have the cluster heads been changed, but also the number of the nodes in each cluster has been changed. As shown in Table 1, when 10% of the nodes are moved, the No. of the nodes which move within the cluster are 1, 75, 12, 88 and 31, and the No. of the nodes which move between different clusters are 17, 98, 7, 67 and 73. Although the cluster heads and their number of nodes both have been changed after the movements, all the selected cluster heads avoid the border position of the monitoring area, and the numbers of nodes in each cluster are relatively similar. This can keep the topology of network stable in a mobile environment. Compared with the classical clustering algorithm LEACH, the proposed TCM algorithm can avoid the cluster heads from being selected in the edge of the monitoring region. This proves that the TCM method can realize a relative uniform clustering, and improve the network stability.

**Table 1:** The topology change result after the movement of the nodes.

		A	B	C	D	E	F
Topology without movement	No. of cluster head	Base Station	36	29	22	57	92
	Number of nodes	16	17	16	17	13	16
Topology after movement	No. of cluster head	Base Station	41	72	50	81	82
	Number of nodes	15	15	18	15	15	17

The above A, B, C, D, E, F in Table 1 are the cluster numbers corresponding to A, B, C, D, E, F in Figure 6 and Figure 7.

### 4.2. Amount of Data Transmitted 

**Figure 8:**
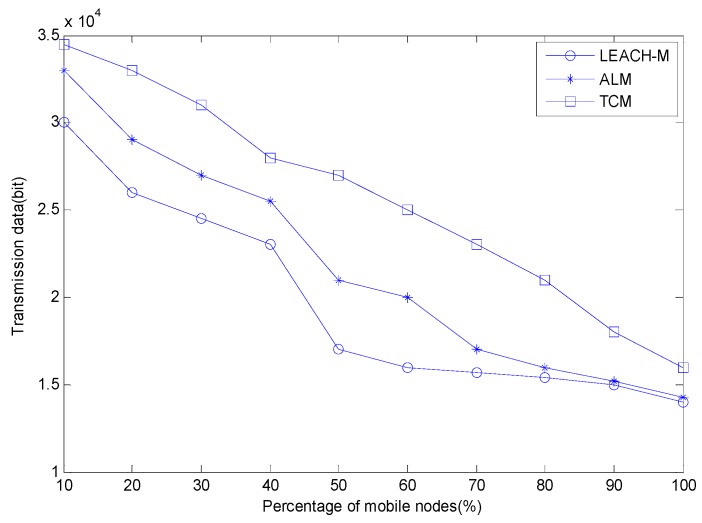
The relationship of transmission data and the percentage of mobile nodes.

The relationship between transmission data and the percentage of mobile nodes is shown in Figure 8.

In Figure 8, as the percentage of mobile nodes increases, the data transmission decreases. The method proposed in this paper has an improvement of 14.3%–58.8% compared to LEACH-M and 10%–23.5% compared to ALM, which indicates the proposed method has better data transmission performance. The reason is that we use a soft handover manner just like in mobile communication to keep communicating with the previous cluster when the nodes just move into the new cluster. The nodes will not break the link with the previous cluster until the moving nodes establish a stable communication link with the new cluster. Although this manner inevitably increases the energy consumption when the percentage of moving nodes increases, it guarantees the transmission performance of the network. The ALM algorithm will adaptively expand the communication range of mobile nodes when the moving nodes move between the clusters, but it does not maintain the links with previous cluster heads in this process. Although it can more quickly recover the link between the moving nodes and the new cluster head than LEACH-M, its data transmission quantity is still worse than that of the proposed TCM method.

### 4.3. Network Energy Consumption and Security

**Figure 9:**
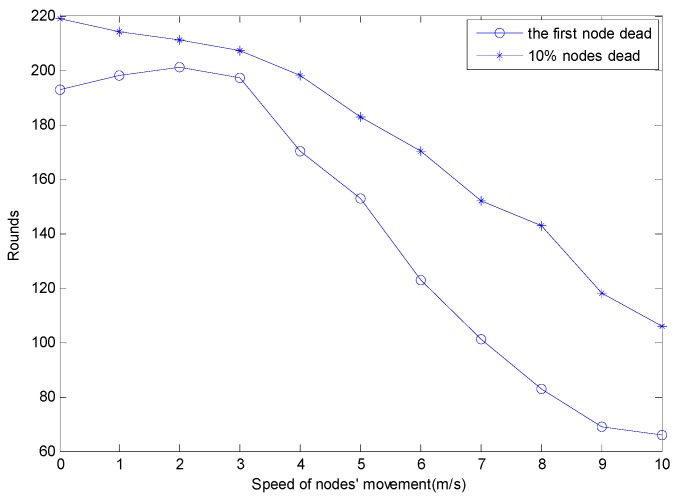
The influence of nodes’ speed on the network death time.

The influence of nodes’ speed on the death time of the network is shown in Figure 9.

**Figure 10:**
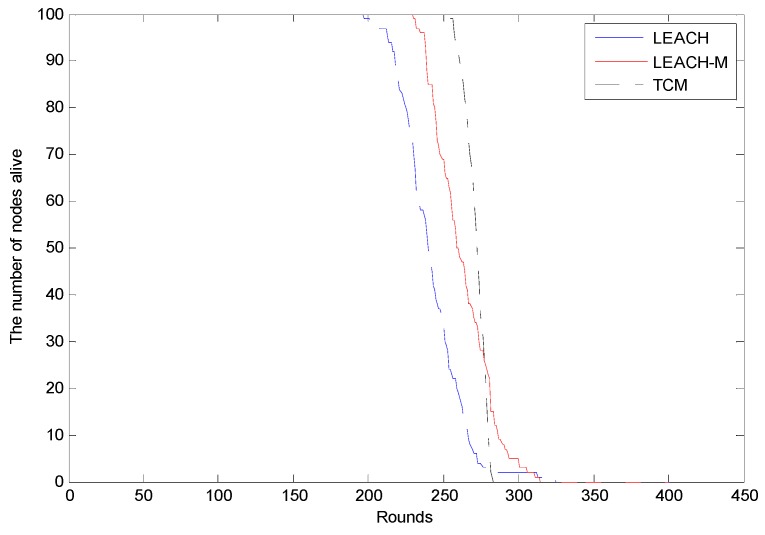
Comparison of the nodes’ survival numbers for three schemes.

From Figure 9, it can be seen that as the speed of nodes increases in the range of 0–10 m/s, the round number of the first dead node’s appearance will increase in the beginning, and then it will decrease in most of the interval. The round number of the 10% dead nodes appearing will decrease as the moving speed increases. The reason is that when the speed of movement of the nodes is too fast, the topology changes violently, and the percentage of the nodes moving across the boundary of clusters will increase. In the topology maintenance stage, the nodes which move across the boundary of the clusters need to maintain links with the new and the previous cluster head simultaneously, so the energy consumption for maintaining the safety of the link will be larger. If we define the round of the first dead node’s appearance as the lifetime of the network, the best speed of movement of the nodes is 2 m/s, and at this speed the first dead node appears in round 201. A comparison of the survival nodes’ numbers for the three schemes is shown in Figure 10.

Figure 10 shows the first dead node in the three methods occurs in the 197th, 229th and 255th round, respectively. The proposed method can extend the lifetime of network as much as 58 rounds more than the LEACH method and 26 rounds more than the LEACH-M method. In addition, as Figure 10 shows, the black line falls faster than the red line and the blue line, that is to say the proposed TCM can make the energy more uniform during clustering. In the proposed method, we consider the residual energy of cluster heads, the distance from the sensor nodes to cluster heads and mobility of the nodes comprehensively in the clustering stage, and this can ensure the selected cluster heads satisfy that the condition that sum of distances between the sensor nodes and cluster heads is minimal and the energy left is maximal. 

In the topology maintenance phase, the TDMA time plot assignment and communication mechanism of soft handover are adopted. TDMA time slot assignment makes the nodes only work in their own time plots, and this wake mechanism can effectively decrease the energy consumption and extend the network lifetime. When entering a new cluster, the moving nodes will be authenticated by the digital signature based on ECC. By this step, the attacks from malicious nodes could be effectively detected. Without this mutual identity authentication, malicious nodes could pretend to be legitimate ones and communicate with the nodes to get critical information about the network, which could seriously affect the network security. After confirming the legitimacy, the moving nodes can send requests to join the clusters, and the nodes will maintain communication links with the previous cluster head and the new cluster head simultaneously until they establishes a stable link with the new cluster. This mechanism can ensure the moving nodes crossing the boundary quickly recover communication, and ensure the continuity of information transmission. 

## 5. Conclusions

The classical clustering methods like the LEACH algorithm, LEACH-M algorithm and ALM algorithm have some disadvantages for MWSNs, such as large energy consumption, low topology stability and low data transmission efficiency. The proposed method can solve these problems. In the topology establishment phase, the previous research only considers one or two factors, and in our proposal we optimize some factors comprehensively with multi-objective optimization to ensure the energy left is maximal and the movement is minimal. In addition, the cluster heads are selected to ensure the sum of the distances from member nodes to the cluster head is minimal, which can improve the balance and stability of the network, and also avoid energy holes in the network and extend its lifetime. In the topology maintenance phase, the TDMA time slot assignment wake mechanism and the soft handover communication mechanism are introduced to decrease the energy consumption, ensure the legality of mobile nodes’ identity and decrease the attacks from malicious nodes. 
